# Developing a curriculum framework for global health in family medicine: emerging principles, competencies, and educational approaches

**DOI:** 10.1186/1472-6920-11-46

**Published:** 2011-07-22

**Authors:** Lynda Redwood-Campbell, Barry Pakes, Katherine Rouleau, Colla J MacDonald, Neil Arya, Eva Purkey, Karen Schultz, Reena Dhatt, Briana Wilson, Abdullahel Hadi, Kevin Pottie

**Affiliations:** 1 Department of Family Medicine, McMaster University, 175 Longwood Road South, Hamilton, L8S 1A4 Canada; 2 Dalla Lana School of Public Health, University of Toronto, 155 College Street, Health Science Building, 6th floor, Toronto, M5T 3M7 Canada; 3 Department of Family Medicine, St. Michael's University of Toronto, 30 Bond Street, Toronto, M5B 1W8 Canada; 4 Faculty of Education, University of Ottawa, Lamoureux Hall (LMX), 145 Jean-Jacques- Lussier Private, Ottawa, K1N 6N5 Canada; 5 Office of Global Health, Schulich School of Medicine & Dentistry, 1151 Richmond Street. The University of Western Ontario, London, N6A 5C1 Canada; 6 Department of Family Medicine, Queen's University, 220 Bagot Street, Kingston, K7L 5E9 Canada; 7 Department of Family Medicine, Northern Ontario School of Medicine, 1813 Lasalle Boulevard Sudbury, P3A 2A3 Canada; 8 Independent member, 128 Emerald Street S, Hamilton, L8N 2a5, Canada; 9 Institute of Population Health, University of Ottawa, 1 Stewart Street, Ottawa, K1N 6N5 Canada; 10 Institute of Population Health, Department of Family Medicine, University of Ottawa, 1 Stewart Street, Ottawa, K1N 6N5 Canada

## Abstract

**Background:**

Recognizing the growing demand from medical students and residents for more comprehensive global health training, and the paucity of explicit curricula on such issues, global health and curriculum experts from the six Ontario Family Medicine Residency Programs worked together to design a framework for global health curricula in family medicine training programs.

**Methods:**

A working group comprised of global health educators from Ontario's six medical schools conducted a scoping review of global health curricula, competencies, and pedagogical approaches. The working group then hosted a full day meeting, inviting experts in education, clinical care, family medicine and public health, and developed a consensus process and draft framework to design global health curricula. Through a series of weekly teleconferences over the next six months, the framework was revised and used to guide the identification of enabling global health competencies (behaviours, skills and attitudes) for Canadian Family Medicine training.

**Results:**

The main outcome was an evidence-informed interactive framework http://globalhealth.ennovativesolution.com/ to provide a shared foundation to guide the design, delivery and evaluation of global health education programs for Ontario's family medicine residency programs. The curriculum framework blended a definition and mission for global health training, core values and principles, global health competencies aligning with the Canadian Medical Education Directives for Specialists (CanMEDS) competencies, and key learning approaches. The framework guided the development of subsequent enabling competencies.

**Conclusions:**

The shared curriculum framework can support the design, delivery and evaluation of global health curriculum in Canada and around the world, lay the foundation for research and development, provide consistency across programmes, and support the creation of learning and evaluation tools to align with the framework. The process used to develop this framework can be applied to other aspects of residency curriculum development.

## Background

Medical trainees in high-income countries have a growing interest to practice medicine with vulnerable and marginalized populations in both domestic and international settings [1]. Ethical and sustained practice throughout the global village demands an understanding of the complex forces that influence the health of individuals and populations [2]. It also requires practical skills that enable effective practice in resource-limited settings including articulating roles as advocates and professionals both locally and globally [3]. The Paris Declaration on Aid Effectiveness (2005) emphasizes the need for working together and focusing on results [4]. While core content competencies are emerging [5], there is a need for standardized global health curriculum [6] and strategies to address these dynamic trainee needs [7].

Global health emphasizes the central importance of health equity and the need for interdisciplinary collaborative actions to address health inequities [8]. Learning to address issues that transcend national boundaries and directly or indirectly affect health can inform all levels of health professional training [9] and can also help trainees effectively engage marginalized communities, the poor, the underserviced, immigrants and refugees, and persons living in remote and isolated communities in their native countries [10].

Inherent in global health practice is ethical community engagement and appreciating the relationship between health status and health systems in high, middle and low income countries [11]. In addition to the potential spread of communicable diseases across borders [12], the populations of all countries are affected by a web of interdependent determinants of health such as the biophysical environment, macroeconomics, social structures and politics [13]. Finally, global health practice without adequate attention to developing competencies could result in harm to patients and their communities.

The purpose of this initiative was to develop an explicit and comprehensive Global Health education framework and to identify enabling competencies to guide curriculum for all Ontario Family Medicine postgraduate training programs. Enabling competencies specify the behaviours, skills and attitudes that learners must demonstrate to reach the Canadian Medical Education Directives for Specialists (CanMEDS) competencies. The CanMEDS framework is a medical education guide to the essential competencies that physicians need to have for improved patient care. In addition, we felt our collaborative efforts would reduce redundancies in global health curriculum development and research, take advantage of existing strengths and expertise in various programs, resources, and research and result in greater credibility and productivity to the relatively new discipline of global health education.

## Methods

Building on recently developed theoretical frameworks from other medical education initiatives [14,15], our aim was to identify and incorporate key educational elements to achieve desired global health education outcomes. We used an evidence-informed approach [16], defining key questions, searching literature and existing curricula and using a consensus approach to design our process and framework. The intensive process of developing a framework occurred between January and June 2010.

### Working Group

Representatives from Ontario's six Family Medicine postgraduate training programmes participated in the one day meeting at an off campus site. Leaders in global health were contacted, and with the assistance of programme chairs, these leaders identified additional key faculty members and residents who they felt could add value to the process. The core group consisted of 10 faculty members (with family medicine, global health, and/or medical education expertise), 2 education consultants, a research assistant, project manager, and an administrative assistant.

### Scoping Review

A scoping review of the literature was performed focussing on theoretical frameworks and core competencies for global health education and a Medline review (2000-2010) on published reports discussing service learning and mentorship as core components for global health education. Databases included Medline, ERIC, Embase, Cochrane Library, and EconLit Birme/VHL, EQUIDAD and Google Scholar. We selected literature based on relevance and quality as suggested by a description of methods and focused the synthesis on frameworks, service learning and mentorship. Details of the search strategy are available in a technical document [17].

### Consensus Process

Nine Canadian Family Medicine global health experts and 2 experts in medical curriculum development from McMaster University, University of Ottawa, University of Toronto, Queen's University, the Northern Ontario School of Medicine and The University of Western Ontario met to develop a collaboration and develop a framework for global health curriculum development for family medicine. Building on the evidence from the scoping review (discussed in the results section of this paper), the group followed a process which included debate and consensus on a global health definition for family medicine, mission statement, key guiding principles, values, important learning approaches and finally, a curriculum framework. Notes were taken and the session was videotaped and reviewed by LRC for consistency.

Teleconferences: The working group met for weekly (2 hour) teleconferences for two months before and four months following the one-day consensus meeting. Background work and sharing of documents was performed through email and telephone calls. The framework was used as a guide to develop enabling competencies for global health for Canadian Family Medicine and these were based on the structure of CanMEDS -Family Medicine roles (a framework that adapts the specialist core competency model) [18,19] (See Table 1 for steps used to develop this systematic framework).

**Table 1:** Steps to develop a systematic framework for Global Health Family Medicine

1. Identification of experts from several disciplines, with invitations to join a working group.
2. Comprehensive review of relevant literature focused on global health curriculum.
3. Consultations with key educators and content experts external to the working group.
4. Working group brainstorming sessions with exchange of relevant data.
5. Consensus meeting to agree on methods and factors (values and principles, competency domains, and learning strategies), to draft a framework and use it to develop specific global health enabling competencies.
6. Additional evidence synthesis
7. Serial teleconferences to draft competencies, concepts, principles and the framework
8. Review and approval of final framework and review documents

## Results

The scoping review identified several global health competency initiatives [5,20-22] but no comprehensive curriculum frameworks for curriculum development in family medicine. The review identified a comprehensive framework for health education [23], and a framework for public health competencies [24]. In addition, mentoring, service learning and self-reflection were identified as key learning approaches for global health education [25-27]. Finally our technical document provided a qualitative synthesis of published articles related to mentorship and service learning.

The background work identified four essential tasks that were fundamental in drafting a curriculum framework: 1. Developing a mission statement. 2. Defining the nature and scope of global health as it applies to family medicine. 3. Delineating the core values and principles relevant to global health education and practice in family medicine. 4. Determining unique learning approaches for global health education relevant for a curriculum framework.

### Creating a Mission Statement

The objectives of the mission statement were twofold: 1. Assist faculty and staff in developing, implementing and evaluating global health content, teaching/learning strategies, venues and evaluation tools to meet the needs of family residents in a changing global environment. 2. Provide learners (as consumers) with a concise goal and manage expectations of GH programs.

Using a theoretical paper on global health as a spring board for ideas [8], we used a consensus process to arrive at the following mission statement. The mission statement developed for global health education in Family Medicine training was; 'The family physician's role in global health is to achieve health equity through advocating and fostering relationships with individuals and populations addressing the broad determinants of health.'

### Defining Global Health for Family Medicine

Using a recently published definition of global health [8], the group debated and discussed the essential elements of a definition of global health specific for Family Medicine. The group proposed a working definition for global health as a foundation for further curriculum development. The consensus working definition of Global Health for Family Medicine developed was 'Global Health is an area of education, research and practice that places priority on improving health and achieving equity in health for all people worldwide.'

### Identifying Core Values and Principles for Global Health and Family Medicine Education

Building upon the work conducted by the University of Toronto Department of Family and Community Medicine [28,29], we identified core values and principles that could provide an essential background context for core competencies and evaluation and that are shared by all global health family physicians. The eleven defining values and principles for global health were: social justice, sustainability, reciprocity, respect, honesty and openness, humility, responsiveness and accountability, equity, and solidarity. (See table 2 for definitions)

**Table 2:** Values and Principles underlying Global Health and Family Medicine Education

**Social justice **- fair and impartial access to the benefits of society including the right to health
**Sustainability- **living and working within the limits of available physical, natural and social resources in ways that allow living systems to thrive in perpetuity.
**Reciprocity - **multidirectional sharing and exchange of experience and knowledge among collaborating partners
**Respect - **for the history, context, values and cultures of communities with whom we engage
**Honesty and openness **- in planning and implementation of all collaborations
**Humility **- in recognizing our own values, biases, limitations and abilities
**Responsiveness and accountability- **to students and faculty and diverse communities with whom we are involved
**Equity **- promoting the just distribution of resources and access, especially with respect to marginalized and vulnerable groups
**Solidarity - **ensuring that objectives are aligned with those of the communities with which we are working

### Determining unique learning approaches for global health training relevant for an education framework

Four pedagogical domains from the literature were identified by the working group as essential to the development and implementation of the curriculum: curriculum delivery, mentorship models, practice opportunities/models and evaluation/assessment. (Table 3 lists the components)

**Table 3:** Components of Global Health Education Framework

1. Definition of Global Health
2. Mission of Global Health
3. Principles and Values
4. Global Health Competencies
5. Curriculum delivery
6. Mentorship
7. Service Learning and Practice Settings
8. Evaluation

**Figure 1:**
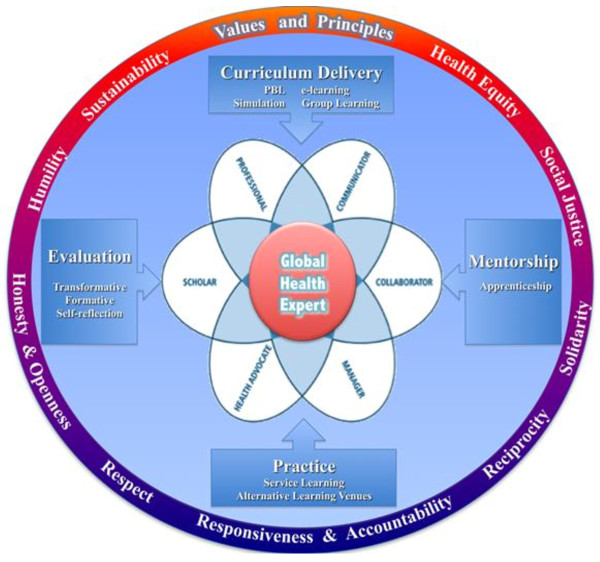
**Framework for Global Health Education in Postgraduate Family Medicine Training **Available online at: http://globalhealth.ennovativesolution.com^©^The Ontario Global Health Family Medicine Curriculum Working Group, (2010) [17]

This educational framework (figure 1) provides a visual representation of the values and principles, core competencies, and learning methods to support the development of skills, curriculum, and leadership for Family Medicine training programs. Each CanMEDS-oriented core competency identifies knowledge and skills necessary to provide effective primary care to diverse patients and their communities. Gaining proficiency in these competencies takes place through a balance of theoretical and practical learning experiences. Theoretical content may be delivered through a variety of methods including: seminars, problem based learning sessions, small study groups, individual readings, simulations and eLearning modules. Practical knowledge and skills are acquired through experiential electives (service learning) in Canada and/or abroad. Through the course of their theoretical and practical training, and in particular through formal mentorship, it is expected that trainees will appreciate and demonstrate attitudes and behaviours consistent with the values and principles outlined in the framework. An understanding of all elements of the framework, including values and principles will be enhanced through regular and structured self-reflection and evaluation.

### Global health competencies

The following descriptions align global health competencies with CanMEDS-Family Medicine roles and provide a context for enabling competencies. Detailed competencies for global health are outlined in the technical document[17].

#### Professional

Working in an environment with different cultural, medical and social norms, often with additional roles and authority, can pose significant challenges for setting boundaries and maintaining professionalism. Unfamiliar disease states and resource limitations can pose complex and irresolvable ethical dilemmas. For these reasons, when working in a setting without well- defined roles and responsibilities practitioners must see themselves as ambassadors for the medical profession by acting within their knowledge and skill level, and developing enhanced skills in identifying and resolving ethical conflicts at the individual and population levels.

#### Communicator

The role of communicator in the Global Health setting requires that family physicians be able to build rapport with all patients including those from varied socioeconomic and cultural groups. It is essential that primary care providers are able to effectively communicate despite social, economic, cultural and language barriers and are aware of interpretation services in their communities. Practitioners should also be adept at dispensing important health information to appropriate officials, as well as to the public through various media outlets.

#### Collaborator

In the global arena, the team caring for a patient may include health care professionals with scopes of practice that differ from those found in Canada. Local culture may influence the ways in which team members relate and work together beyond the immediate and most obvious implications of scopes of practice. Gender and local politics for example, may play a role in the construction and function of the health care team. Similarly, the patient's social support might extend beyond the boundaries of what is considered the family in Canada, possibly involving various members of the extended family, clan, tribe, ethnic or religious kins. Understanding the organization of the local health care system, at the national, regional and local levels and including both the formal and informal elements is essential for those working in global health. In order to collaborate effectively, the global health practitioner must determine his or her role both as an individual physician and as a representative of a Canadian or foreign organization. Developing relationships with the various local levels of governments and community organizations is essential to achieving true collaboration.

#### Advocate

The duty of health advocacy extends beyond advocacy within the healthcare system to advocacy for improvement in the determinants of health for the individuals and populations they serve. The Global Health practitioner recognizes the significant influence physicians may bring to bear on a variety of issues related directly and indirectly to health, and works with professionals from other fields to advocate for disadvantaged populations and lobby bodies and governments to improve health for all.

#### Medical (Global Health) Expert

Family Medicine expertise includes knowledge, skills and attitudes to effectively provide care for individual patients from a wide range of cultural, geographic, and socioeconomic status groups. This care may be provided in a wide variety of settings, some of which may include shelters, addiction clinics, mobile clinics, First Nations, Métis, and Inuit communities, and international settings. The ability to provide effective care for these diverse populations and in these diverse settings will mean the physician will need to build upon the CanMEDS competencies, extrapolate and apply evidence to diverse patients and populations. Relevant factors include: how prevalence and incidence of disease may differ for an unfamiliar population, how cultural beliefs and health-related behaviours may differ, how physician preferences and attitudes toward these populations may differ, and how patient's preferences may differ. Rather than being an expert in all populations, the global health expert will use a systematic process to assess uniqueness of a population, and to consider local resources to provide the most feasible and effective care.

#### Scholar

The practice of family medicine in a global health context includes humanitarian assistance, health system development and reform, health equity, population health, immigrant and refugee health, inner city health, indigenous health. In the creation, dissemination, application and translation of knowledge family physicians working in global health need to master and apply methods and strategies that are adapted to the unique needs of the patients and communities with which they work.

#### Manager

As Global Health practitioners, family physicians recognize themselves as participants in varied traditional and non-traditional international health systems. They may also interact with and lead intergovernmental or non-governmental agencies, service providers or health- related organizations. They recognize the differing resource-allocation needs of different populations and settings. Whether they are at home or abroad, family physicians work towards quality improvement (efficiency, good management, and respect for persons) of all health systems and health professionals with which they are involved.

## Discussion

This global health curriculum framework is unique in that it incorporates not only core competencies for global health, but also contextual values and principles, and dynamic learning approaches. In addition, it provides a shared foundation to support collaboration and exchange during the development of global health curriculum for Ontario's six family medicine residency programmes. Curriculum frameworks can provide structure for evaluation and can lead to higher quality curriculum [30]. This global health curriculum framework provides a structure similar to other health education, health promotion [26,31-35] and research initiatives [36]. The development process could also be applied to the development of other curricula within Family Medicine as well as to other postgraduate training programs.

With some notable exceptions [6,21,36], most of the published literature on global health and medical education has focused on undergraduate medial training programs [22,37,38]. Evert et al. (2007) write about the introduction of global health into family medicine training programs and identify the importance of champions for global health and tailoring training programs to best fit the available resources [36]. They also identify the importance of service learning and mentoring and identify the financial, supervisory, and sustainability challenges of global health programs.

This global health curriculum framework does not aim to define global health curricular content, but rather to serve as a dynamic curriculum development road map for educators. It was developed for family medicine educators in Ontario and its relevance for other groups remains to be determined. Modifications to the framework are expected as the framework continues to evolve. Integrating perspectives from educators in other health disciplines and educators from both low and higher income countries will serve to enhance both the framework's utility in Canada and its potential impact to guide global health education worldwide. Evidence for best practice learning methods for global health continues to emerge, however the quality of evidence to date remains limited.

## Conclusions

A shared process and curriculum framework provides a foundation to design, deliver, and evaluate global health curriculum to meet the needs of faculty, residents and patients. It ultimately seeks to enhance the effectiveness of those working in the area of global health. This achievement is the first step to lay the foundation for more research and development, provide consistency across programmes, consensus and network building and tool creation for global health in family medicine curricula in Ontario. Next steps will include seeking national family medicine endorsement of the framework and working with international organizations to align with other global health education initiatives. It is hoped that our collaborative efforts will reduce redundant global health curriculum development and research initiatives, utilize strengths and expertise in various Ontario family medicine programs and result in greater credibility and productivity to the relatively new discipline of global health education.

## Competing interests

The authors declare that they have no competing interests.

## Authors' contributions

This paper was an output of the Ontario Global Health Family medicine curriculum working group. This paper is truly the result of a combined group effort. All authors have made substantial contributions to conception and design, analysis and interpretation of data, have been involved in drafting the manuscript or revising it critically for important intellectual content; and have given final approval of the version to be published. LRC was the project initiator, received funding and lead the working group. KP acted as the co-lead for the group. LRC and KP completed final edits and decisions for the paper. LRC, KP, KR, BP contributed as equal authors. CJM was integral in developing the framework model, providing expertise to the group with respect to educational framework models and contributing this to the paper. NA provided important intellectual content and consistent involvement. KS contributed significantly to the mapping development and EP on the specific curriculum competencies. AH acted as the research assistant and contributed additional content. BW and RD contributed important intellectual content to the process, content and provided final approval.

## Pre-publication history

The pre-publication history for this paper can be accessed here:

http://www.biomedcentral.com/1472-6920/11/46/prepub
